# Alterations of contralesional hippocampal subfield volumes and relations to cognitive functions in patients with unilateral stroke

**DOI:** 10.1002/brb3.3645

**Published:** 2024-08-12

**Authors:** Juan‐Juan Lu, Xiang‐Xin Xing, Jiao Qu, Jia‐Jia Wu, Mou‐Xiong Zheng, Xu‐Yun Hua, Jian‐Guang Xu

**Affiliations:** ^1^ School of Rehabilitation Science Shanghai University of Traditional Chinese Medicine Shanghai China; ^2^ Department of Rehabilitation Medicine, Yueyang Hospital of Integrated Traditional Chinese and Western Medicine Shanghai University of Traditional Chinese Medicine Shanghai China; ^3^ Department of Radiology Shanghai Songjiang District Central Hospital Shanghai China; ^4^ Department of Traumatology and Orthopedics, Yueyang Hospital of Integrated Traditional Chinese and Western Medicine Shanghai University of Traditional Chinese Medicine Shanghai China; ^5^ Engineering Research Center of Traditional Chinese Medicine Intelligent Rehabilitation Ministry of Education Shanghai China

**Keywords:** cognitive impairment, hippocampal subfields, lateralization, magnetic resonance imaging, stroke, volume

## Abstract

**Background:**

The volumes of the hippocampal subfields are related to poststroke cognitive dysfunctions. However, it remains unclear whether contralesional hippocampal subfield volume contributes to cognitive impairment. This study aimed to investigate the volumetric differences in the contralesional hippocampal subfields between patients with left and right hemisphere strokes (LHS/RHS). Additionally, correlations between contralesional hippocampal subfield volumes and clinical outcomes were explored.

**Methods:**

Fourteen LHS (13 males, 52.57 ± 7.10 years), 13 RHS (11 males, 51.23 ± 15.23 years), and 18 healthy controls (11 males, 46.94 ± 12.74 years) were enrolled. Contralesional global and regional hippocampal volumes were obtained with T1‐weighted images. Correlations between contralesional hippocampal subfield volumes and clinical outcomes, including the Montreal Cognitive Assessment (MoCA) and Mini‐Mental State Examination (MMSE), were analyzed. Bonferroni correction was applied for multiple comparisons.

**Results:**

Significant reductions were found in contralesional hippocampal as a whole (adjusted *p* = .011) and its subfield volumes, including the hippocampal tail (adjusted *p* = .005), cornu ammonis 1 (CA1) (adjusted *p* = .002), molecular layer (ML) (adjusted *p* = .004), granule cell and ML of the dentate gyrus (GC‐ML‐DG) (adjusted *p* = .015), CA3 (adjusted *p* = .009), and CA4 (adjusted *p* = .014) in the RHS group compared to the LHS group. MoCA and MMSE had positive correlations with volumes of contralesional hippocampal tail (*p *= .015, *r* = .771; *p* = .017, *r* = .763) and fimbria (*p* = .020, *r* = .750; *p* = .019, *r* = .753) in the LHS group, and CA3 (*p* = .007, *r* = .857; *p* = .009, *r* = .838) in the RHS group, respectively.

**Conclusion:**

Unilateral stroke caused volumetric differences in different hippocampal subfields contralesionally, which correlated to cognitive impairment. RHS leads to greater volumetric reduction in the whole contralesional hippocampus and specific subfields (hippocampal tail, CA1, ML, GC‐ML‐DG, CA3, and CA4) compared to LHS. These changes are correlated with cognitive impairments, potentially due to disrupted neural pathways and interhemispheric communication.

## Graphical abstract

This study suggests volumetric differences in the contralesional hippocampal subfields between patients with left hemisphere stroke (LHS) and those with right hemisphere stroke (RHS). Specifically, RHS resulted in greater volumetric reduction in the whole contralesional hippocampus and specific subfields (hippocampal tail, CA1, ML, GC‐ML‐DG, CA3, and CA4), and these changes are correlated with poststroke cognitive impairments. The study concluded that unilateral stroke caused volumetric differences in different hippocampal subfields contralesionally, potentially due to disrupted neural pathways and interhemispheric communication.

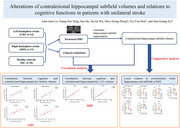

## 1 INTRODUCTION

Approximately 20%–80% stroke survivors have cognitive deficits, resulting in poor ability of self‐care, communication, and social interaction (Chaurasia et al., 2019). At present, there is no pharmacotherapy approved for poststroke cognitive impairments (PSCI), and the benefits of cognitive rehabilitation are not yet supported by robust evidence due to methodological limitations in existing research (Quinn et al., 2021; Rost et al., 2022). Besides, the underlying mechanisms of PSCI still remain unclear (Quinn et al., 2021). Therefore, it is critically important to understand the brain changes associated with PSCI in order to develop specific treatments that can prevent or delay the onset of PSCI and further deterioration of cognitive function.

It is known that brain atrophy may have predated and predicted an impaired cognitive status, and of different brain structures, a measure of hippocampal volume alone or jointly both could enhance the reliability of predicting effects (Jack et al., 2005). Hippocampal atrophy is also a core characteristic of vascular cognitive impairment (Hosseini et al., 2017). For instance, poststroke patients usually exhibit hippocampal atrophy, which has been positively linked to the development of cognitive impairment (Barber et al., 2021; Kliper et al., 2013). In contrast, one neuroimaging study found no relationship between hippocampal atrophy and PSCI (Stebbins et al., 2008). Conflicting results may be due to the limitations of considering hippocampus as a whole. Hippocampus, a complex structure, comprises various interconnected subfields, which may be related with specialized functions and display atrophy that is related to a specific disease by the method for automated segmentation of hippocampal subfields (Wong et al., 2021; Zhao et al., 2021). It is widely acknowledged that hippocampal subfield volumes are more specific and sensitive markers of cognitive performance compared to global hippocampal volume (Uribe et al., 2018). Numerous studies have targeted hippocampal subfields and have established the close correlations between global cognitive performance and specific hippocampal subfields, such as cornu ammonis areas (CA) 1, molecular layer (ML), granule cell layer of the dentate gyrus (GC‐ML‐DG), presubiculum, and subiculum (Doran et al., 2023; Yasuda et al., 2022). Furthermore, volumetric alterations of relatively small subfields would make less contribution to those of the whole hippocampus, that is, even though there is no correlation between hippocampal volumes and cognitive functions, it does not mean any subfield involvement in cognition. Therefore, regarding hippocampus as a whole during correlation analysis limited the sensitivity for diagnosis of cognitive impairment. Global cognition is organized in a large‐scale cognitive network of multiple relevant circuits, depending on the coordination of various brain regions and their interconnections (Khodagholy et al., 2022). Damage to these regions, especially key regions, can greatly impact the global cognitive performance. Notably, the hippocampus serves as a key hub in cognitive networks, with its functions supported by the specific roles of its distinct subfields (Battaglia et al., 2011). Existing evidence showed that hippocampal subfields displayed a selective vulnerability to brain injury (Frankowski et al., 2019; Gemmell et al., 2012). Considering that each subfield uniquely contributes to the overall function of the hippocampus, structural deficits in any hippocampal subfield can affect not only specific cognitive domains but also the coordinated interactions of cognitive circuits centered on the hippocampus, thereby leading to widespread impacts on global cognitive function. Hence, we focus on the volumetric changes in hippocampal subregions and their contributions to cognitive impairment following stroke to identify specific subfields significantly linked to PSCI. These findings will provide neuroimaging evidence to further elucidate the underlying pathological mechanisms of PSCI and offer specific targets for drug development and neuromodulation therapies. Moreover, pinpointing the hippocampal subfields associated with PSCI enables precise interventions in these areas, optimizing therapeutic outcomes. The atrophy observed in these subfields may assist in identifying poststroke patients at a higher risk of cognitive decline, facilitating early intervention and treatment.

In addition, we noted that one study found an equal decrease of hippocampal volumes in bilateral hemispheres (Khlif et al., 2019), and another study even reported a significantly lower volume of hippocampus in the contralesional hemisphere than that in the ipsilesional hemisphere after ischemic stroke (Haque et al., 2019). Based on previous evidence that the significant correlations between ipsilesional hippocampal subfields and PSCI have been reported (Chen et al., 2016; Khlif et al., 2021), it is reasonable to presume the potential role of contralesional hippocampal volumes in cognitive functions in poststroke patients. To our knowledge, present studies were primarily concerned with the volumetric alternations of the ipsilesional hippocampus and its link to PSCI, and the effect of contralesional hippocampal volume has not been systematically evaluated. In this context, we emphasized the exploration of roles of volumes of contralesional hippocampal subfields in poststroke cognitive functions.

Multiple lines of evidence have suggested that left hippocampus plays a dominant role in verbal memory, whereas the right hippocampus is more involved in nonverbal memory (Powell et al., 2005; Richardson et al., 2004). This finding implies that volumes of contralesional hippocampus, even hippocampal subfields, may play quite different roles in cognitive functions between patients with left hemisphere stroke (LHS) and right hemisphere stroke (RHS).

The present study aimed to explore volumetric changes of contralesional hippocampal subfields in patients with LHS and RHS compared to healthy individuals. Moreover, we performed partial correlation analysis to explore the relations between contralesional hippocampal subfield volumes and clinical evaluation of cognitive functions in patients with LHS and RHS, respectively.

## 2 MATERIALS AND METHODS

### 2.1 Participants

Structural magnetic resonance images (MRIs) and clinical evaluations in this experiment were obtained from 14 patients with LHS, 13 patients with RHS, and 18 healthy controls (HCs). Inclusion criteria for patients were as follows: (1) first onset of unilateral ischemic or hemorrhagic stroke during the past 3–12 months (Shi et al., 2017; Yin et al., 2013); (2) 18–70 years old, no gender limitation; (3) right‐handed; (4) tolerance to neurological and MRI examinations. Aged between 18 and 70 years old, right‐handed individuals, who had no history of stroke, were included in the HC group. Participants were excluded based on the following criteria: (1) with structural abnormalities visible in MRI caused by other diseases; (2) with contraindications to MRI; (3) with neurological or psychiatric impairments; (4) being unable to understand instructions (Mini‐Mental State Examination [MMSE] < 22) (Ma et al., 2022); (5) with a prior history of significant cognitive decline (based on participant and informant history and medical records), and dementia family history (Akinyemi et al., 2017; Brodtmann et al., 2020).

Patients were recruited from the outpatient department of Yueyang Hospital of Integrated Traditional Chinese and Western Medicine, Shanghai University of Traditional Chinese Medicine. HCs were from the community.

### 2.2 Clinical evaluations

Montreal Cognitive Assessment (MoCA) and MMSE evaluate global cognitive performance and impaired cognition are defined as scores of MoCA <26 (Ma et al., 2022) and MMSE <27 (Wang et al., 2021).

Modified Barthel Index (MBI) is a measure of functional independence, and a higher score represents greater independence (Shah et al., 1989).

Berg Balance Scale (BBS) is a 14‐item scale assessing the ability to maintain balance, with a higher score indicating stability of balance (Wei et al., 2019).

Fugl–Meyer Assessment of upper extremity (FMA‐UE) is applied to assess the degree of synergistic movements in the affected upper extremity, with higher scores indicating better performance (Lin et al., 2022).

### 2.3 MRI data acquisition and image processing

T1 scanning was conducted on a MAGNETOM Verio 3T MRI scanner (Siemens Healthcare), and its parameters were set as follows: flip angle = 9°, repetition time = 1900 ms, inversion time = 900 ms, echo time = 2.93 ms, voxel size = 1.0 × 1.0 × 1.0 mm^3^.

**FIGURE 1:**
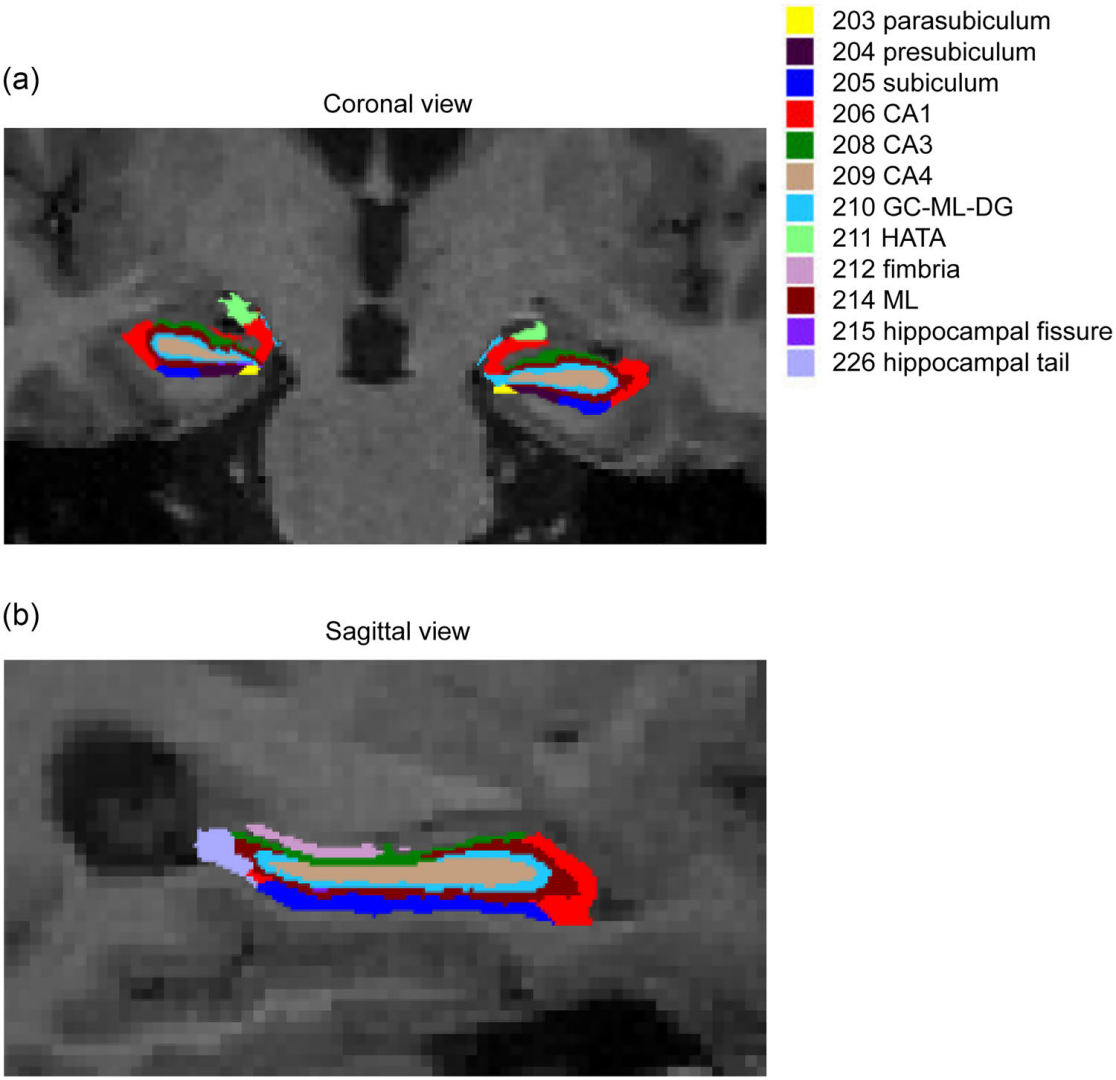
Visualization of hippocampal subregion segmentation. Hippocampal segmentation with labeled subfields in a healthy participant was shown in the coronal (a) and sagittal views (b). CA1, cornu ammonis 1; GC‐ML‐DG, granule cell and molecular layer of the dentate gyrus; HATA, hippocampal–amygdaloid transition area; ML, molecular layer.

The automatic hippocampal subfield segmentation of T1‐weighted scans was performed using the image‐processing pipeline of the FreeSurfer software (Version 6.0, Martinos Center for Biomedical Imaging), as depicted in Figure 1. The hippocampal volumes were then measured globally and regionally, including hippocampal tail, subiculum, cornu ammonis 1 (CA1), hippocampal fissure, presubiculum, parasubiculum, ML, GC‐ML‐DG, CA3, CA4, fimbria, and hippocampal‐amygdaloid transition area.

### 2.4 Statistical analysis

Data analysis was performed with SPSS ver. 25.0 software (SPSS). The mean ± standard deviation was used to present numerical variables, and *n* (percentage) was used to represent categorical variables. Analysis of covariance (ANCOVA) or Kruskal–Wallis test was performed to determine the difference of continuous variables among three groups, followed by the post hoc comparison. Comparisons in categorical variables were performed by Chi‐Square (*χ* ^2^) test. Clinical evaluations between LHS and RHS groups were compared using the Wilcoxon rank sum test.

The comparisons in brain volumes and global and regional hippocampal volumes between LHS and RHS groups were conducted using ANCOVA or Kruskal–Wallis test, followed by Bonferroni correction for post hoc multiple comparisons. All statistical tests were two‐tailed, and statistics were regarded as significant if *p* value was <.05.

Controlling for age, sex, time since stroke, hypertension, and diabetes mellitus, Spearman's partial correlation analysis between volumes of the whole hippocampus and all the hippocampal subfields and clinical evaluations between LHS and RHS were performed via a MATLAB R2013b platform (The MathWorks Inc.), respectively. In order to visualize partial correlation results, the residuals of hippocampal volumes and scores of clinical evaluations were computed, respectively, and scatterplots were then generated using GraphPad Prism version 8.0.1 (GraphPad Prism) (Hackett et al., 2021; Perret et al., 2021). *p* Threshold of .05 was used to label statistical significance.

## 3 RESULTS

### 3.1 General characteristics and clinical data

**FIGURE 2:**
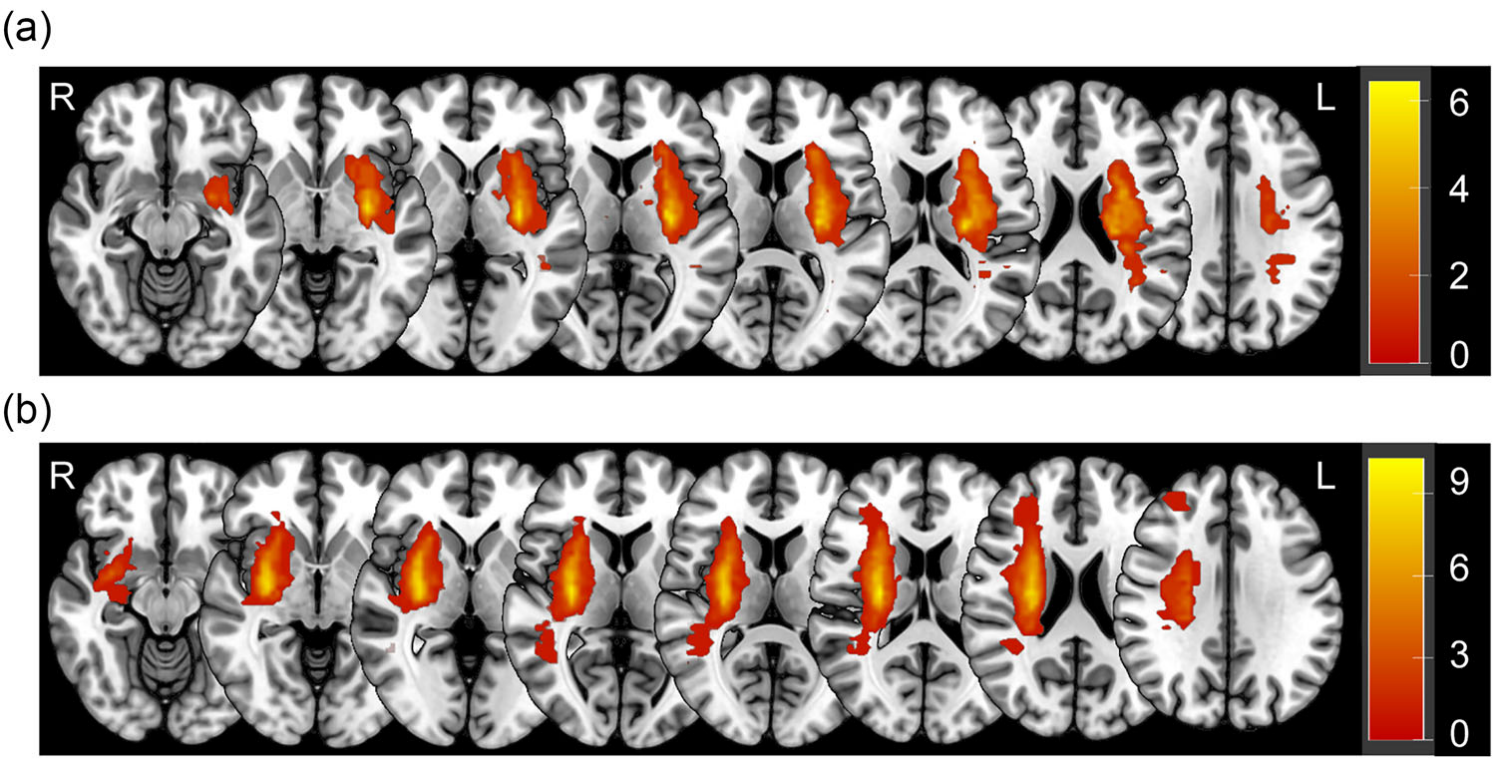
Overlay maps of cerebral lesions in patients with stroke. Overlay maps showed the distribution of cerebral lesions in the patients with left hemisphere stroke (a) and right hemisphere stroke (b). Color bar denotes the number of overlapping lesions, and warmer color indicates a higher level of overlap. L, left hemisphere; R, right hemisphere.

In this study, a total of 45 participants were enrolled, including 14 left‐lesioned and 13 right‐lesioned stroke patients, and 18 HCs. Among 27 stroke patients, 74% (*n* = 20) and 30% (*n* = 8) of them had a history of hypertension and diabetes mellitus, respectively. They were classified into large artery atherosclerosis (*n* = 15) and small artery occlusion categories (*n* = 12) based on the Trial of ORG 10172 in Acute Stroke Treatment (TOAST) classification (Adams et al., 1993). Cerebral lesions of these stroke patients were identified as punctate (*n* = 8) and territorial (*n* = 19) lesions (Jeon et al., 2018). The lesion overlay maps for each group are shown in Figure 2. No significant difference was observed in general characteristics among LHS, RHS, and HC groups (Table 1). Additionally, significant differences were found in the total brain volume (TBV) between the RHS and HC groups (adjusted *p* = .038) and in the TBV/total intracranial volume between the LHS and HC groups (adjusted *p* < .001), as well as between the RHS and HC groups (adjusted *p* < .001). In terms of clinical evaluations, significant difference was found in BBS scores (*p *= .046) between LHS and RHS groups. Comparisons between these two groups exhibited no difference in MBI, FMA‐UE, MMSE, or MoCA scores.

**TABLE 1:** Baseline data and clinical evaluations for poststroke patients and healthy controls.

Characteristics		LHS (*n* = 14)	RHS (*n* = 13)	HC (*n* = 18)	*p*‐Value
Age (years)	M (SD)	52.57 ± 7.10	51.23 ± 15.23	46.94 ± 12.74	.415^a^
	Min–Max	40–65	29–68	30–66	
	Median (Q1, Q3)	53 (47.25–58.25)	55 (35.50–65.00)	45 (35.00–59.25)	
Gender, male (%)		13 (93%)	11 (85%)	11 (61%)	.079^b^
Hypertension		10 (71%)	10 (77%)	/	>.999^b^
Diabetes mellitus		4 (29%)	4 (31%)	/	>.999^b^
Lesion laterality		Left	Right	/	/
Time since stroke (months)		6.07 ± 3.13	7.77 ± 3.81	/	.269^c^
Stroke type					
Infarct (%)		12 (86%)	9 (69%)	/	.303^b^
Hemorrhage (%)		2 (14%)	4 (31%)	/	
TIV (mL)		1563.16 ± 135.02	1543.44 ± 131.14	1529.80 ± 171.07	.823^d^
TBV (mL)		1086.32 ± 135.49	1051.98 ± 93.41^*^	1168.88 ± 131.83	.032^d^
TBV/TIV		0.70 ± 0.07^###^	0.68 ± 0.04^***^	0.76 ± 0.02	<.001^a^
MoCA score		26.79 ± 2.99	27.77 ± 2.77	/	.305^c^
MMSE score		26.86 ± 2.91	27.85 ± 2.64	/	.293^c^
MBI score		86.21 ± 15.36	77.85 ± 17.53	/	.093^c^
BBS score		44.86 ± 7.14	33.38 ± 18.37	/	.046^c^
FMA‐UE score		27.29 ± 16.90	19.77 ± 13.33	/	.244^c^

*Note*: Hypertension was diagnosed based on the following criteria: persistent blood pressure (BP) ≥140/90 mmHg.

Diabetes mellitus was diagnosed based on the following criteria: glycohemoglobin A1c (A1c) ≥6.5%, or fasting plasma glucose (FPG) ≥7.0 mmol/l, or 2‐h plasma glucose ≥11.1 mmol/L during an oral glucose tolerance test (OGTT), or a random plasma glucose ≥11.1 mmol/L.

Abbreviations: BBS, Berg Balance Scale; FMA‐UE, Fugl–Meyer Assessment of upper extremity; HC, healthy controls; LHS, left hemisphere stroke; M, mean; Max, maximum; MBI, modified Barthel Index; Min, minimum; MMSE, Mini‐Mental State Examination; MoCA, Montreal Cognitive Assessment; Q25, first (25%) quartile; Q75, third (75%) quartile; RHS, right hemisphere stroke; SD, standard deviation; TBV, total brain volume; TIV, total intracranial volume.

^a^ Kruskal–Wallis test.

^b^ Chi‐Square (*χ* ^2^) test.

^c^ Wilcoxon rank sum test

^d^ Analysis of covariance (ANCOVA).

^###^ *p* < .001, LHS vs. HC.

^*^ *p* < .05.

^***^ *p* < .001, RHS vs. HC.

### 3.2 Comparisons of volumes of contralesional hippocampal subfields

**FIGURE 3:**
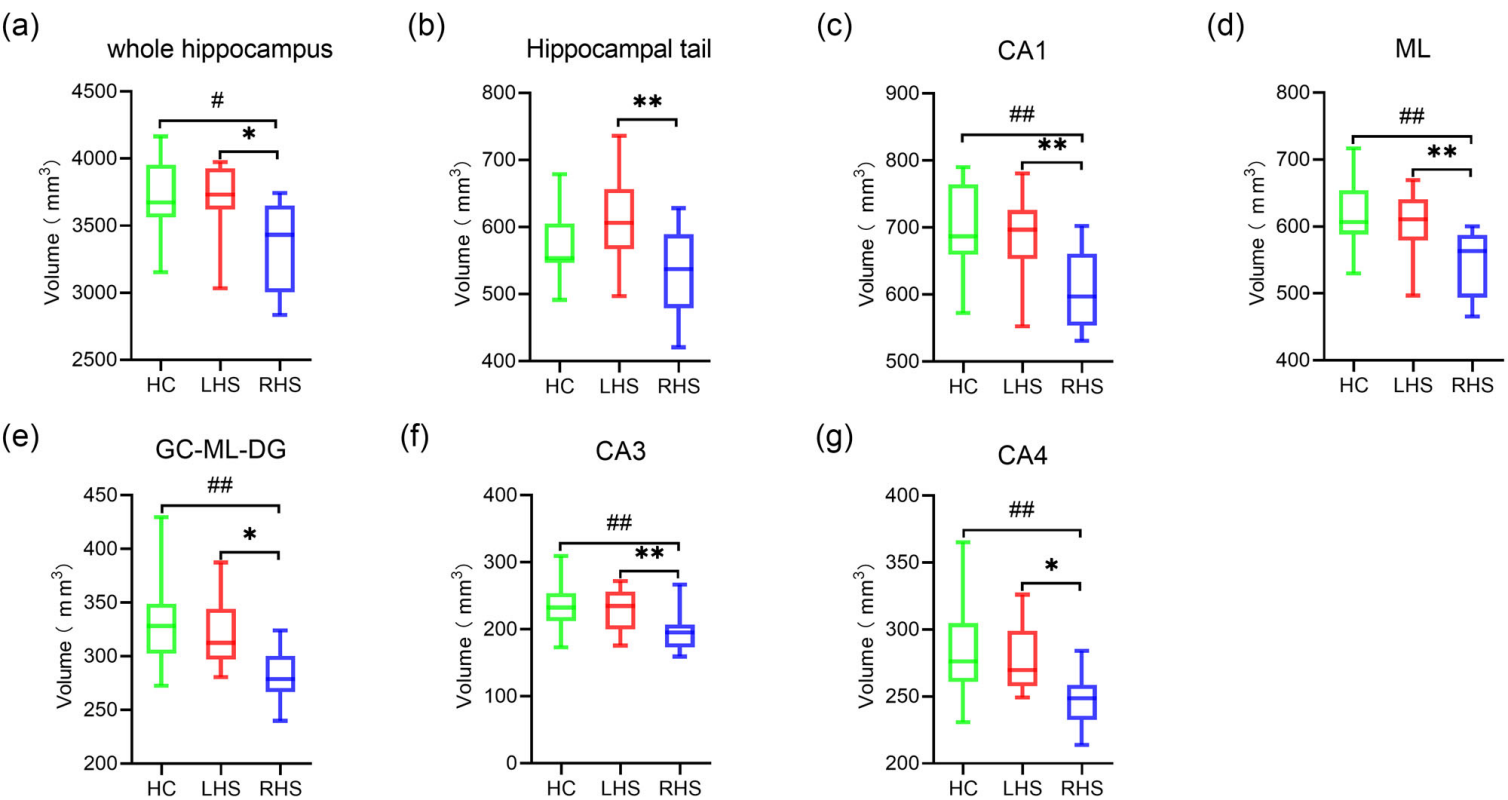
Comparisons of contralesional hippocampal and subfield volumes among patients with left‐lesioned/right‐lesioned stroke and healthy controls. Differences in volumes of the contralesional whole hippocampus (a), hippocampal tail (b), CA1 (c), ML (d), GC‐ML‐DG (e), CA3 (f), and CA4 (g) among the patients with left hemisphere stroke (LHS), patients with right hemisphere stroke (RHS), and healthy controls (HC) were shown. All *p* values were adjusted using the Bonferroni correction. ^#^ *p* < .05, versus HC; ^##^ *p* < .01, versus HC; *p < .05, versus LHS; **p < .01, versus LHS. CA1, cornu ammonis 1; ML, molecular layer; GC‐ML‐DG, granule cell and molecular layer of the dentate gyrus.

The volumes of contralesional hippocampal subfield in patients with LHS were not significantly different from those in the HC group. However, the differences in the volumes of contralesional whole hippocampal (*p *= .006, adjusted *p* = .017) and subfields, including hippocampal tail (*p *= .047, adjusted *p* = .141), CA1 (*p *< .001, adjusted *p* = .001), ML (*p *< .001, adjusted *p* < .001), granule cell molecular layer of the dentate gyrus (GC‐ML‐DG) (*p *< .001, adjusted *p* = .001), CA3 (*p *= .001, adjusted *p* = .002), and CA4 (*p *= .001, adjusted *p* = .002), were significant between the RHS and HC groups. Additionally, RHS patients showed relatively smaller volumes of contralesional whole hippocampus (*p *= .004, adjusted *p* = .011) and subfields, including hippocampal tail (*p *= .002, adjusted *p* = .005), CA1 (*p *= .001, adjusted *p* = .002), ML (*p *= .001, adjusted *p* = .004), GC‐ML‐DG (*p *= .005, adjusted *p* = .015), CA3 (*p *= .003, adjusted *p* = .009), and CA4 (*p *= .005, adjusted *p* = .014) than LHS patients (Figure 3).

### 3.3 Correlations between volumes of contralesional hippocampal subfields and clinical evaluations

#### 3.3.1 Correlation between contralesional hippocampal subfield volumes and clinical evaluations in LHS

**FIGURE 4:**
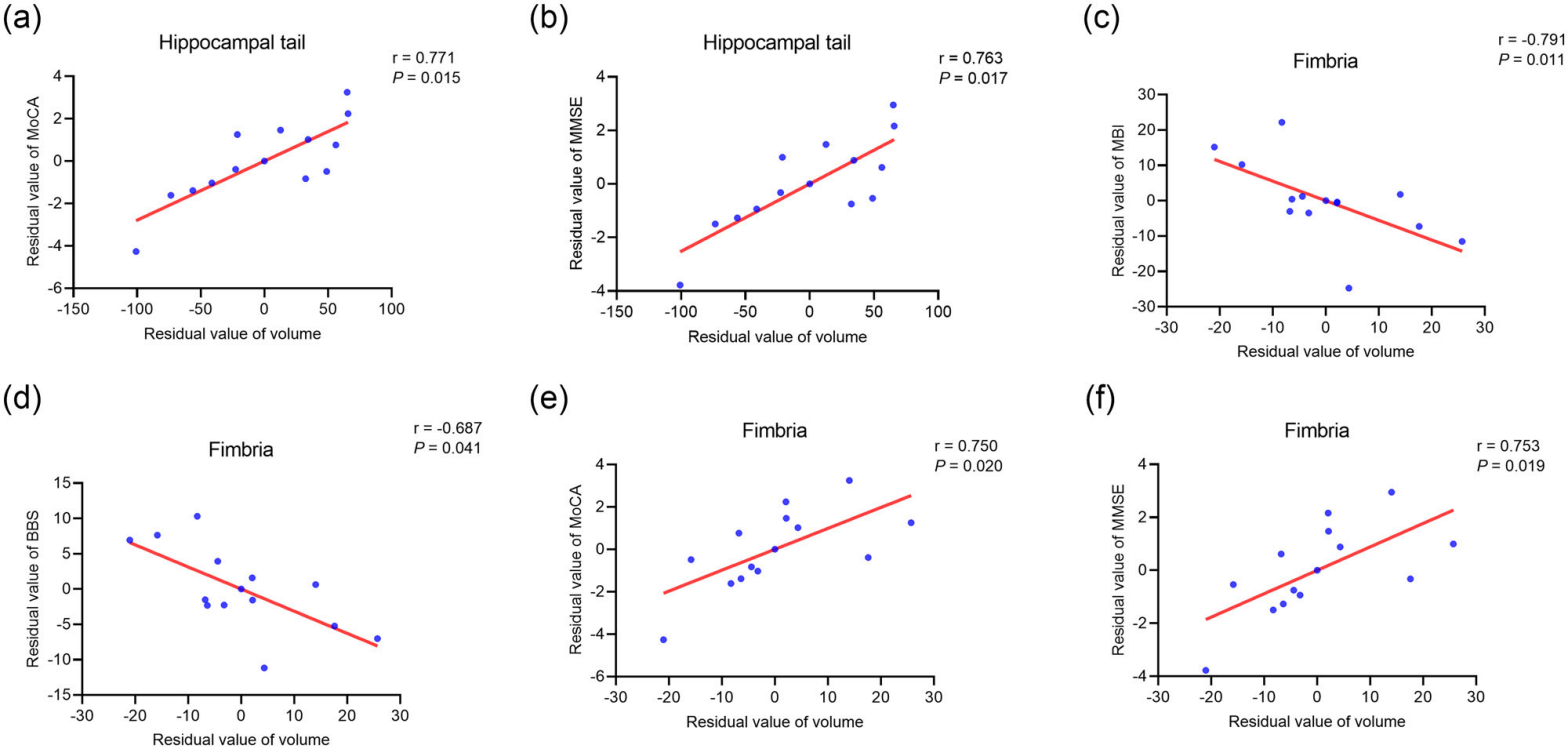
Correlation between contralesional hippocampal subfield volumes and clinical evaluations in patients with left hemisphere stroke. Partial correlations analysis showed positive correlations between volumes of contralesional hippocampal tail and MoCA (a) and MMSE (b), and volumes of contralesional fimbria and MBI (c), BBS (d), MoCA (e), and MMSE (f). BBS, Berg Balance Scale; MMSE, Mini‐Mental State Examination; MBI, Modified Barthel Index; MoCA, Montreal Cognitive Assessment.

After controlling parameters of age, gender, time since stroke, hypertension, and diabetes mellitus, volume‐cognitive correlations were found in the LHS group. Specifically, positive correlations were found between the volume of contralesional hippocampal tail and scores of MoCA (*p *= .015, *r* = .771) and MMSE (*p *= .017, *r* = .763), as well as volumes of contralesional fimbria and scores of MoCA (*p *= .020, *r* = .750) and MMSE (*p *= .019, *r* = .753) (Figure 4). It was also found negative relationships between volumes of contralesional fimbria and MBI (*p *= .011, *r* = −.791) and BBS (*p *= .041, *r* = −.687) (Figure 4).

#### 3.3.2 Correlation between contralesional hippocampal subfield volumes and clinical evaluations in RHS

**FIGURE 5:**
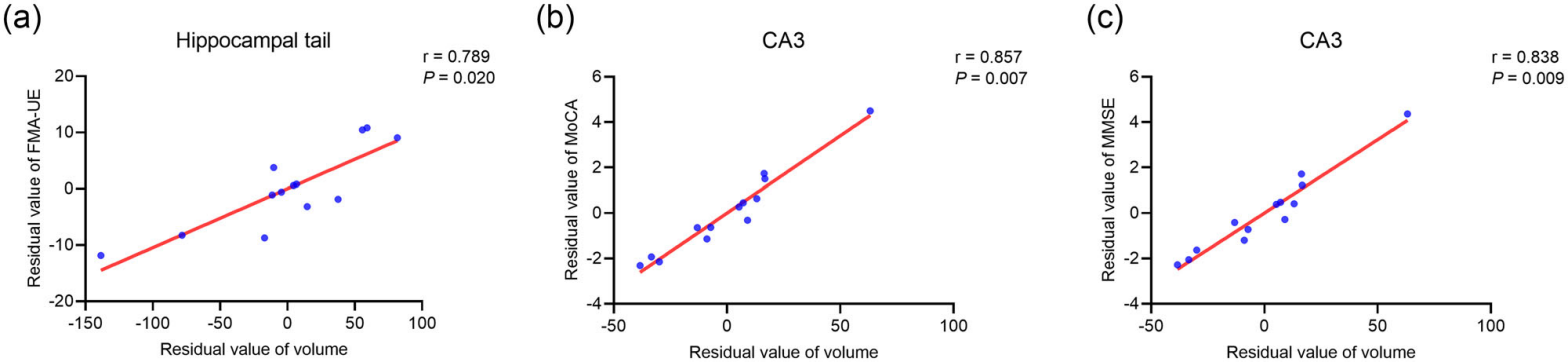
Correlation between contralesional hippocampal subfield volumes and clinical evaluations in patients with right hemisphere stroke. Partial correlation analysis showed positive correlations between volumes of contralesional hippocampal tail and FMA‐UE (a) and volumes of contralesional CA3 and MoCA (b) and MMSE (c). CA3, Cornu ammonis 3; MMSE: Mini‐Mental State Examination; FMA‐UE, Fugl‐Meyer Assessment of Upper Extremity; MoCA: Montreal Cognitive Assessment.

In RHS patients, we found positive correlations between volumes of contralesional hippocampal tail and scores of FMA‐UE (*p *= .020, *r* = .789), as well as volumes of contralesional CA3 and scores of MoCA (*p *= .007, *r* = .857) and MMSE (*p *= .009, *r* = .838) (Figure 5).

## 4 DISCUSSION

Our results showed a reduction of the global and regional hippocampal volumes, including the hippocampal tail, CA1, ML, GC‐ML‐DG, CA3, and CA4, which displayed differences in the RHS group compared to the LHS group. Furthermore, significant correlations between the volumes of contralesional hippocampal subfields and motor or cognitive functions were found in both LHS and RHS patients, but the specific subfields involved differed between the two groups. Based on these findings, it is suggested that volumes of contralesional hippocampal subfields could serve as an underlying mechanism that contributed to PSCI, and this mechanism differs in patients with LHS and RHS.

Our study showed a significant reduction in the volume of whole contralesional hippocampus only in patients with RHS. The study conducted by Brodtmann et al. (2020) found no significant difference in contralesional hippocampal volume in stroke patients at two timepoints (3 and 12 months). We noted that their inclusion criteria were similar to ours (including all subtypes, regardless of cause or site), apart from the grouping by lesion side. This suggests that lesion side might have a certain effect on contralesional hippocampal volume, and grouping by lesion side helps better elucidate hippocampal volumetric alterations following different sides of stroke. Interestingly, stroke patients in their study demonstrated significantly greater percent atrophy in the contralesional hemisphere than that in HCs in both the later and full periods, whereas this effect in the ipsilesional hemisphere appeared only in the early period. One longitudinal neuroimaging study focused on the rates of hippocampal atrophy over 3 years in poststroke patients and reported that patients displayed higher annualized atrophy rates in bilateral hippocampus compared to HCs, which were positively associated with a decline in cognition (Barber et al., 2021). Khlif et al. (2019) also supported this finding that average hippocampal volume was significantly lower in the ischemic stroke group, regardless of the automatic segmentation methods. These findings implied that stroke might cause persistent damage to contralesional hippocampus, and the damage impairing cognitive performance would become apparent over time. Furthermore, whole hippocampus atrophy was greater in the ipsilesional hemisphere than that in the contralesional hemisphere (Aamodt et al., 2022; Khlif et al., 2022), indicating a direct impact of the stroke lesion itself. Contralesional hippocampal damage may be owed to the commissure of fornix and trisynaptic loop in hippocampus (Tang et al., 2012).

Hippocampus is easily damaged by transient ischemia, and the vulnerability varies between hippocampal subfields. Specifically, CA1 is the hippocampal subfield most often affected by transient ischemia (Schmidt‐Kastner & Freund, 1991). Another study found that CA1, CA4, DG, and subiculum were more sensitive to ischemic injury (Li et al., 2016). Consistent with these findings, our study demonstrated that the presence of RHS resulted in significant volumetric changes in contralesional (left) CA1, ML, GC‐ML‐DG, CA3, and CA4. Lower volume in CA1, ML, and GC‐ML‐DG might be due to natural predisposition to neuronal loss (Li et al., 2016). Selective atrophy in hippocampal subfields is a characteristic feature of several dementia‐related diseases. For instance, a declining trend in volumes of bilateral CA1, subiculum, presubiculum, ML, and fimbria was observed with the progression of Alzheimer's disease (AD) (Zhao et al., 2019), whereas Parkinson's disease patients were found to have smaller volumes in some different hippocampal subfields, such as bilateral CA2/3 and CA4 (Xu et al., 2020). These findings suggested distinct patterns of volumetric alterations across hippocampal subfields in various diseases. To gain further insight into the involvement of contralesional hippocampus, we refer to the discovery from Khlif et al. (2022) that volumes in the contralesional hippocampal tail and presubiculum reduced sharply over 9 months due to stroke. However, the effects of the lesion side were not explored in their study. In general, studies on volumetric alternations of the contralesional hippocampal subfields are limited. Compared to HC, significant differences were found in the left hippocampal field volumes in patients with RHS, whereas there was no significant difference in the right hippocampal field volumes in patients with LHS. Contralesional hippocampal volume in patients with RHS was significantly lower than that in patients with LHS, which survived Bonferroni correction, suggesting that left hippocampus might have a greater susceptibility to injury. Some scholars proposed one possible explanation that anatomical asymmetry between lateral hippocampal volumes (R > L) might predispose the left hippocampus to a much greater vulnerability to disease pathology than the right hippocampus (Kannappan et al., 2022; Khlif et al., 2019). The asymmetries in the atrophy patterns observed in AD populations have been linked to increased expression of β‐amyloid in the left hemisphere (Tsai et al., 2009). Anatomically, information transmission within the hippocampal subfields follows the classic trisynaptic pathway: from the entorhinal cortex to the DG GCs via the perforant path, then to the CA3 pyramidal cells through the mossy fibers, and finally to the CA1 pyramidal cells via the Schaffer collaterals (Andersen, 2007). In addition to the ipsilateral associational projections, commissural projections are crucial for inter‐hippocampal communication, such as the CA3‐to‐CA1 and DG‐to‐DG projections (Swanson et al., 1981; Zimmer, 1971). It has been reported that inter‐hippocampal projection is more susceptible to inflammation, which was regarded as a primary mechanism in poststroke cerebral damage (Wu et al., 2020). Volumetric reductions in the ipsilateral hippocampus subfields following stroke may suggest underlying widespread neuronal loss and synaptic loss, which could further propagate to the contralateral hippocampal subfields through inter‐hippocampal projections. Moreover, we believe that there were some additional factors that may have potentially contributed to volumetric differences in hippocampus between these two groups (Homayouni et al., 2023; Sawyer et al., 2020). Age effects on subfield volumes varied, with older adults exhibiting more pronounced atrophy in CA1–2, CA3, and DG (Daugherty et al., 2016). Additionally, the age‐CA1 volume association was enhanced in females (Homayouni et al., 2023). Besides, comorbidities like hypertension and diabetes mellitus could selectively induce volumetric reductions in hippocampal regions, such as hippocampal tail and DG (Dong et al., 2019; Li et al., 2020). Therefore, even though these factors do not show significant between‐group differences, we have accounted for their effects in subsequent data analyses to ensure the reliability of our study's findings.

One of our primary findings was that volumes of poststroke contralesional hippocampal tail were positively correlated with cognitive functions in the LHS group and sensorimotor functions of the affected upper extremity in the RHS group. A previous study showed that patients with LHS tended to have better verbal memory, mainly immediate and delayed recall, when volumes in their hippocampal tail were larger (Khlif et al., 2021). Apart from structural alternations in hippocampal tail related to cognitive decline, decreased resting‐state functional connectivity among bilateral hippocampal tail, right medial prefrontal cortex, and left temporoparietal junction also showed negative correlations with cognitive performance (Liang et al., 2020). These studies have revealed significant correlations between alterations in the hippocampal tail's structure and functions and cognitive functions, suggesting a potential role of the hippocampal tail in cognitive functions. In contrast, the involvement of the hippocampal tail in poststroke motor performance has seldom been conclusively demonstrated in existing studies. In addition, some studies have suggested that the volumes of hippocampal tail may play a role in the diagnosis of major depressive disorder and predicting the relieved effect of antidepressant medication (Maller et al., 2018; Tai et al., 2021). It made sense to do more work exploring the role of volumes of contralesional hippocampal tail in poststroke depression.

In the LHS group, correlations between volumes of contralesional fimbria and activities of daily living (ADL), balance, and cognitive functions were also observed. Fimbria is a bunch of white matter fibers, which then become the fornix after detaching from hippocampus. It is primarily involved in memory storage (Helbing & Angenstein, 2020) and spatial memory processing (Dahmani et al., 2019), which shows a great impact on ADL and balance performance (Kanchan et al., 2018; Rogge et al., 2017). Meanwhile, as a structural bridge connecting hippocampus to other brain structures, its integrity is important for maintaining hippocampal function in memory (Wendelken et al., 2015). Deep brain stimulation (DBS) regarded fimbria‐fornix as the potential target for cognitive and memory deficits, and this intervention could ameliorate learning and memory capability (Lozano et al., 2016) and also improve emotional and social performance (Mao et al., 2018).

Finally, we also found a positive correlation between volumes of contralesional CA3 and cognitive functions in the RHS group. CA3 aids in implementing autoassociation memory via establishing associations between spatial and other, including object, representations to form event or episodic memories (Rolls, 2010). Therefore, CA3 was shown to be involved in encoding and early retrieval processes in memory (Hasselmo et al., 2005). Research by Carlesimo et al. (2015) has linked volumes of CA2/3 to immediate and delayed memory performance in mild cognitive dysfunction and AD patients. In a previous study of the volume changes in the ipsilesional (left) hippocampal subfields at 3 months after LHS, researchers found that larger CA2/3 volumes indicated better delayed memory (Khlif et al., 2021), which was consistent with our finding in the contralesional (left) side in RHS group.

In regard to methodology, it is noteworthy that high field MRI and multimodal imaging are superior in the field of hippocampal subfields. Although researchers have shown that the intrarater reliability of the 3 T study was higher than that of the 7 T study, and image artifacts brought by 7 T MRI might affect hippocampal subfield segmentation, it is undeniable that 7 T MRI demonstrates the superiority in accurate segmentation of hippocampal subfields (Sämann et al., 2022; Wisse et al., 2016). High field MRI exhibits a greater tissue contrast, signal‐to‐noise ratio, and higher spatial resolution, facilitating more detailed and clearer visualization of hippocampal subfields (Perera Molligoda Arachchige & Garner, 2023; Wisse et al., 2016). Compared to low 3 T MRI, 7 T MRI makes it feasible that small volumetric differences and subtle changes within hippocampal subfields can be detected in the disease state (Giuliano et al., 2017; Peixoto‐Santos et al., 2018). Hence, the utilization of 3 T MRI in the current study may present a potential limitation. Multimodality imaging is rapidly advancing; it helps comprehensively understand the functions of the hippocampal subfield. For example, a combination of MRI with [^18^F]fluoro‐deoxyglucose‐positron emission tomography (PET) simultaneously can precisely delineate hippocampal subfields that depict metabolic differences (Carlson et al., 2020). An integrated electroencephalography (EEG)–MRI study investigated the relations between brain rhythmicity and hippocampal atrophy, not yet specific to hippocampal subfields (Moretti et al., 2007). Currently, EEG‐PET‐MRI is recognized for its capacity to describe the functional states of the brain from different perspectives under the same physiological conditions, holding promise for its impact on the diagnosis, and treatment of neuropsychiatric disorders (Arachchige, 2023a, 2023b). However, there is a lack of trimodal neuroimaging research in the exploration of hippocampal subfield alterations under pathological conditions. Despite existing technical and practical limitations of multimodal imaging tools, more research is encouraged to address these issues and promote the advancement of this field.

There are still limitations that need attention. First, our sample size of patients was relatively small, and a larger sample is needed to further support our findings. Second, considering that volumes of hippocampal subfields are related to many domains of cognitive functions, such as memory, recognition, and execution, comprehensive tests should be included in the future study. Third, we did not separately study the effects of lesion locations on volumes of hippocampal subfields. Further studies on the lesion‐specific effects on hippocampal subfields and cognitive functions are required. Last but not least, although we conducted partial correlation analysis controlling for key covariates, other variates such as education and occupational skill level, currently smoking, drinking, and the presence of diseases like hypertension and diabetes mellitus can also affect cognitive performance and volumes of hippocampal subfield volumes (Blom et al., 2020; Castilla‐Guerra, 2022; Jiang et al., 2019; Orsholits et al., 2022; Zhang et al., 2021). It is imperative to conduct comprehensive examinations in participants and incorporate these factors into future analyses. Considering the heterogeneity in clinical demographics (e.g., age, gender, education level), comorbidities (e.g., hypertension, diabetes mellitus), and stroke characteristics (e.g., lesion locations and sizes), a larger study population stratified by these variables would provide a clearer elucidation of the impact on contralateral hippocampal subfield volumes.

## 5 CONCLUSIONS

In summary, we showed that patients with LHS and RHS exhibited volumetric alterations in different contralesional hippocampal subfields. The hippocampal subfield contralateral to the lesion side may variously respond to the lesion in nature, which was reflected by volumetric differences. Correlations between contralesional hippocampal subfields and cognitive performance differed in LHS and RHS survivors. This may suggest a crucial role of contralesional hippocampal subfields in PSCI, as well as a varied contribution of volumes of contralesional hippocampal subfields to cognitive impairment in patients with LHS and RHS. These specific associations implied that subfield volumes were to be a useful biomarker for PSCI, and current rehabilitation methods, such as DBS, could selectively target at contralesional hippocampal subfield, considering the lesion side, to promote poststroke cognitive recovery. There is a need to develop future interventions to induce structural changes in hippocampal subfields for PSCI. This study first described volumetric differences in contralateral hippocampal subfields after stroke by considering the lateralization of the lesion.

## AUTHOR CONTRIBUTIONS

**Juan‐Juan Lu**: Formal analysis; writing—original draft; visualization. **Xiang‐Xin Xing**: Data curation; investigation. **Jiao Qu**: Resources; investigation. **Jia‐Jia Wu**: Methodology; funding acquisition. **Mou‐Xiong Zheng**: Conceptualization; writing—review and editing; funding acquisition. **Xu‐Yun Hua**: Conceptualization; supervision; funding acquisition. **Jian‐Guang Xu**: Conceptualization; project administration; funding acquisition.

## CONFLICT OF INTEREST STATEMENT

The authors declare no conflicts of interest.

### PEER REVIEW

The peer review history for this article is available at https://publons.com/publon/10.1002/brb3.3645.

## Data Availability

The data supporting this study's findings are available from the corresponding author upon reasonable request.
